# Neuroanatomical organization: the palato-pharyngeal complex as a sensory-motor hub

**DOI:** 10.3389/fnana.2026.1779669

**Published:** 2026-03-30

**Authors:** Yong-Shin Hong, Young-Seok Park

**Affiliations:** Department of Oral and Maxillofacial Anatomy, Seoul National University, Seoul, Republic of Korea

**Keywords:** nucleus ambiguus, nucleus tractus solitarius, palato-pharyngeal complex, reflex-volition coupling, trigeminal nerve, vagus nerve

## Abstract

The pharynx has traditionally been described as a musculo-membranous conduit involved in respiration and deglutition. From a neuroanatomical perspective, however, this region also represents a multi-nerve interface where somatic afferents of the trigeminal nerve (V) and visceral afferents of the glossopharyngeal (IX) and vagus (X) nerves converge. In this review, we use the term Palato-Pharyngeal Complex (PPC) to describe this anatomically integrated region and discuss its potential role as a sensory–motor interface associated with brainstem circuits involving the nucleus tractus solitarius (NTS) and nucleus ambiguus (NA). We highlight the coordinated activity of the tensor veli palatini (innervated by V3) and the levator veli palatini (innervated by X) as an example of somatic–visceral motor integration within this region. Building on existing models of brainstem central pattern generators, we discuss a hierarchical control framework in which brainstem circuits may be modulated by supranuclear influences from cortical and limbic systems. Within this context, we introduce the concept of a Reflex–Volition Coupling (RVC) zone as a possible functional interface between reflexive brainstem rhythms and volitional or affective control. By integrating comparative and connectomic perspectives, this review outlines how branchial motor circuitry may support a range of behaviors including airway protection, vocalization, and speech. Together, these observations suggest that the PPC may represent an anatomically strategic interface linking peripheral cranial nerve afferents with brainstem integrative networks involved in arousal and behavioral coordination. This framework provides a basis for future studies exploring how oropharyngeal sensory pathways interact with central neural circuits.

## Introduction

Traditionally, the pharynx has been characterized merely as a passive musculo-membranous conduit connecting the nasal and oral cavities to the esophagus and larynx, primarily serving the mechanical functions of respiration and deglutition (Gray et al., 2021; Lang, 1995). However, from a neuroanatomical perspective, this region can be considered a multi-nerve convergence zone. Specifically, the Palato-Pharyngeal Complex (PPC)—comprising the soft palate and the upper pharynx—represents a unique anatomical intersection where the somatic sensory-motor systems of the trigeminal nerve (CN V) and the visceral systems of the glossopharyngeal (CN IX) and vagus (CN X) nerves densely converge (Doty, 2015; Mu and Sanders, 2000). This convergence suggests that the PPC may function not only as a biological passageway but also as a sensory–motor integration interface (Matsuo and Palmer, 2008).

The neurophysiological basis of this integration lies within brainstem circuitry, specifically the loop between the Nucleus Tractus Solitarius (NTS), which processes sensory afferents, and the Nucleus Ambiguus (NA), which generates motor efferents (Jean, 2001; Bieger, 1984). This NTS-NA circuit forms a key component of the brainstem network involved in generating rhythmic motor patterns for reflexes such as swallowing, coughing, and airway protection (Feldman and Del Negro, 2006; Smith et al., 2007). Crucially, this Central Pattern Generator (CPG) facilitates the integration of multiple neural systems; for instance, the opening of the Eustachian tube requires the synergistic coordination of the tensor veli palatini (innervated by the mandibular nerve, V3) and the levator veli palatini (innervated by the vagus nerve, X) (Ishijima et al., 2002; Heidsieck et al., 2016). This phenomenon indicates that the PPC may act as a junction between somatic and visceral signaling pathways (Matsuo and Palmer, 2009). Contemporary neurophysiological data further suggest that this junction operates through sensory gating mechanisms, where somatic mechanical inputs from the oropharynx can instantly modulate the activation thresholds of visceral airway reflexes, thereby optimizing respiratory-swallowing coordination (Huff et al., 2022).

Across vertebrates, the palato-pharyngeal region participates not only in homeostatic reflexes such as swallowing and airway protection but also in behaviors related to vocal communication and emotional expression. Rodents exhibit laughter-like vocalizations associated with positive affective states, and many bird species use complex vocal signals for social communication. In humans, these evolutionarily conserved sensorimotor circuits are further elaborated to support articulate speech and highly refined vocal behaviors (Rygula et al., 2012; Mooney, 2022).

This expansion implies that volitional and affective commands from the cortex and limbic system are superimposed onto the brainstem’s automatic reflexive circuits (Holstege, 1992; Simonyan, 2014). In this review, we refer to this functional intersection—where survival-oriented reflexes and communication-oriented intentions meet—as the Reflex-Volition Coupling (RVC) zone. The neural balance within this zone is critical; its disruption can lead to pathological conditions like dysphagia or dysphonia (Ertekin and Aydogdu, 2003; Leopold and Kagel, 1997), whereas it’s targeted stimulation may provide opportunities to influence neural plasticity (Hamdy et al., 1998).

Therefore, the present review discusses the pharynx not simply as a static anatomical structure but as a neurophysiological interface that integrates life-supporting reflexes, emotional expression, and vocal behaviors. By examining how the ancestral branchial arch system evolved into this anatomical interface, we discuss a conceptual framework that may inform current neuromodulation research. These observations suggest that the PPC may represent an anatomically accessible site through which brainstem arousal systems and cortical networks could be influenced, which may have implications for therapeutic approaches beyond functional rehabilitation.

## Neuroanatomical architecture: palato-pharyngeal complex as a multi-nerve Nexus

Somatic sensation from the oral and palatal regions is conveyed via the trigeminal nerve (CN V) to the trigeminal sensory nuclear complex, whereas visceral and gustatory afferents from the pharyngeal and laryngeal mucosa reach the NTS through the glossopharyngeal (CN IX) and vagus (CN X) nerves (Jean, 2001; Hayakawa et al., 1998). Recent high-resolution transcriptomic and spatial mapping studies have revealed diverse neuronal populations across brainstem regions, including the trigeminal sensory nuclei and the NTS, providing a molecular framework for understanding their potential functional interactions (Yao et al., 2023). Although these nuclei are anatomically distinct, their juxtaposition within the medulla and their interconnections via the reticular formation establish a region where somatic and visceral sensory information can be integrated (Kleinfeld et al., 2014; Herbert et al., 1990). Furthermore, recent single-cell transcriptomic analyses have unveiled a highly complex molecular Ontology within the NTS, revealing diverse neuronal subtypes capable of coordinating these converging sensory inputs (Gasparini et al., 2024). From an evolutionary perspective, this brainstem-level integration of anatomically distinct sensory streams reflects the evolutionary transformation of the ancestral branchial arch system into a multifunctional cranial interface, as articulated by the “new head” hypothesis. This hypothesis suggests that the vertebrate head evolved as a region associated with expanded sensory and motor integration associated with the emergence of neural crest cells and cranial placodes. These developmental innovations enabled the formation of complex cranial sensory organs and branchial motor circuits that coordinate feeding, respiration, and defensive behaviors. Within this evolutionary framework, the palato-pharyngeal region can be understood as part of a conserved cranial sensorimotor system linking peripheral sensory detection with brainstem motor pattern generation (Gans and Northcutt, 1983; Graham, 2001; Glenn, 2005).

**Figure 1:**
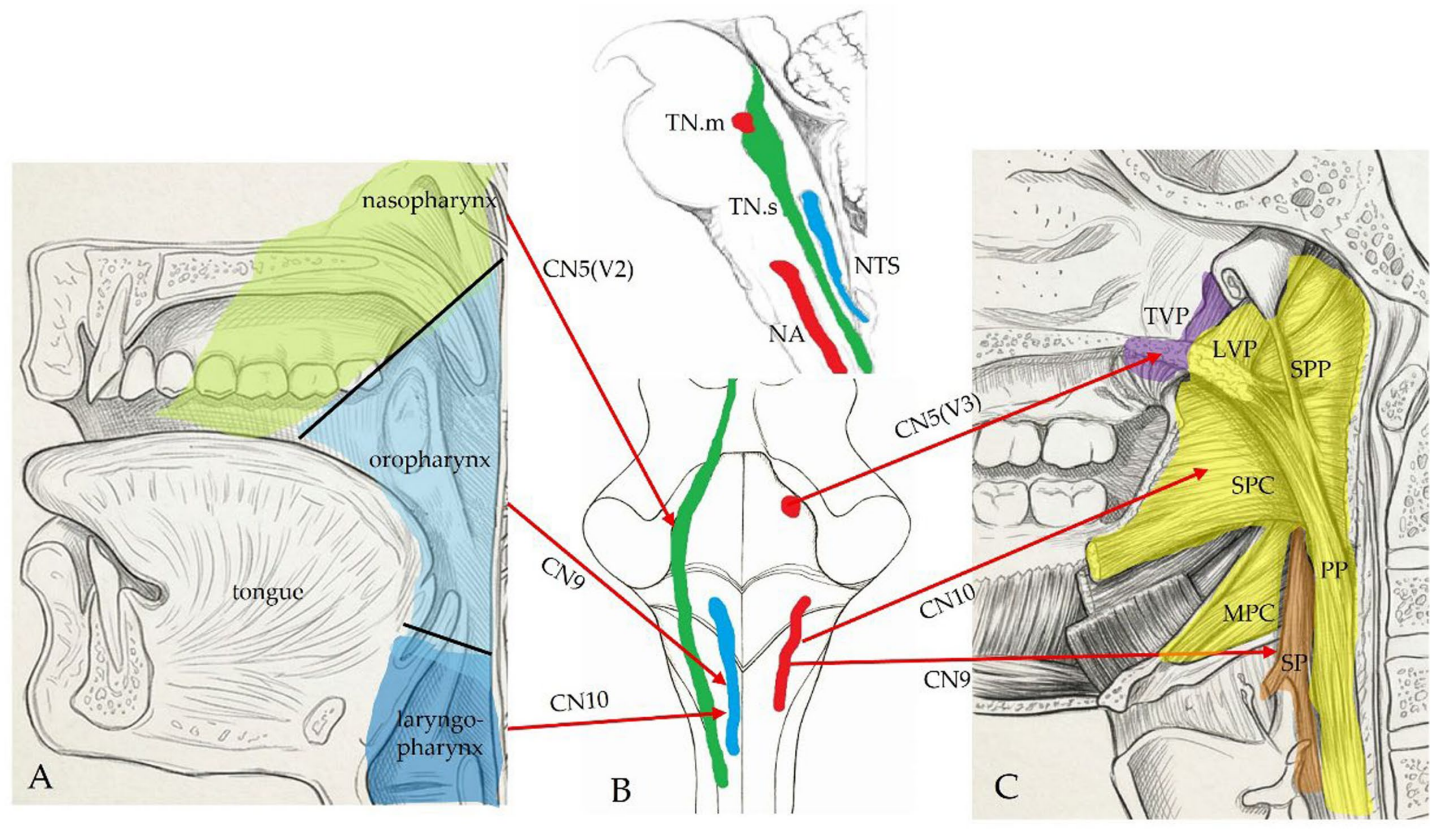
Neuroanatomical organization of the palato-pharyngeal complex (PPC). This schematic illustrates the multi-nerve sensory-motor integration within the PPC. **(A)** Afferent pathways: Peripheral sensory inputs from the palato-pharyngeal region are transmitted via the trigeminal (CN V, specifically V2), glossopharyngeal (CN IX), and vagus (CN X) nerves. These somatic and visceral afferent signals project to and converge upon the trigeminal sensory nuclear complex (including the principal sensory and spinal trigeminal nuclei) and the nucleus tractus solitarius (NTS), respectively. **(B)** Brainstem nuclei topography: A representative view of the brainstem highlighting the spatial arrangement of the critical integrating centers: the trigeminal sensory nucleus, the NTS, the trigeminal motor nucleus, and the nucleus ambiguus (NA). (Top: sagittal section, bottom: posterior view) **(C)** Efferent innervation: Motor projections from the brainstem to the PPC musculature. Efferent fibers originating from the trigeminal motor nucleus (via CN V3) and the NA (via CN X) selectively innervate specific palato-pharyngeal muscles (e.g., tensor veli palatini and levator veli palatini), demonstrating the coordinated somatic-visceral motor outputs essential for proper PPC function. (LVP, levator veli palatini m; MPC, middle pharyngeal constrictor m; PP, palatopharyngeus m; SP, stylopharyngeus m; SPC, superior pharyngeal constrictor m; SPP, salpingopharyngeal m; TN.m (red), trigeminal motor nucleus; TN.s (green), trigeminal sensory nucleus; TVP, tensor veli palatini m).

Rather than representing a peripheral specialization, the emergence of such cranial sensory–motor integration represents a characteristic feature of vertebrate cranial evolution (Gans and Northcutt, 1983; Graham, 2001; Glenn, 2005; Butler, 2000). This architecture allows the PPC to integrate superficial somatic feedback with deep visceral signaling before motor commands are generated by the NA, suggesting a role in sensory integration within brainstem circuits. Recent optogenetic studies suggest that the NTS-NA axis is composed of highly specific, functionally segregated microcircuits that can independently recruit distinct pools of branchial motoneurons (Huff et al., 2023). The motor organization of the PPC provides an example of this coordination, particularly through the synergistic coordination required for human speech and Eustachian tube regulation. This mechanism relies on the precise co-activation of the somatic motor system, represented by the tensor veli palatini (TVP, innervated by CN V3), and the visceral motor system, represented by the levator veli palatini (LVP, innervated by CN X) (Ishijima et al., 2002; Kuehn and Moon, 1998). The collaboration between these two muscles contributes to the acoustic and aerodynamic conditions essential for vocalization. The overall neuroanatomical architecture of the PPC—illustrating the convergence of multi-nerve afferents, the topography of the integrating brainstem nuclei, and the coordinated efferent distribution—is summarized in Figure 1.

Functionally, the LVP functions as a sphincter-like mechanism, elevating the soft palate to close the velopharyngeal port; this action generates the requisite intra-oral air pressure for consonant production and prevents nasal regurgitation (Gyanwali et al., 2016; Perry, 2011). Conversely, the TVP contributes to tension of the soft palate, exerting lateral tension on the soft palate. This tensing action increases the stiffness of the palatal aponeurosis thereby modulating the resonance characteristics and acoustic impedance of the upper airway (Heidsieck et al., 2016; Finkelstein et al., 1990). Consequently, the production of articulate speech can be understood as a phenomenon where somatic motor control (V3) operates together with visceral motor control (X). Such coordinated palatal movements align with established principles of speech and orofacial motor control, in which precise temporal coupling between jaw, velar, and laryngeal systems is required for fluent articulation and mastication (Gracco and Lofqvist, 1994; Lund, 1991). This neuromuscular coordination suggests that the PPC may participate in coordinating multiple cranial nerve systems involved in oropharyngeal motor control, enabling the integration of cortical motor commands through synchronized activity across distinct cranial nerve systems. This functional dichotomy is summarized in Table 1 (Ishijima et al., 2002; Heidsieck et al., 2016; Kuehn and Moon, 1998; Gyanwali et al., 2016; Perry, 2011; Finkelstein et al., 1990).

**Table 1:** Somatic and visceral innervation of the palato-pharyngeal complex.

Neural system	Key nerve	Key muscles	Biomechanical function	Neuromodulation target
Somatic system (ectodermal origin) (Ishijima et al., 2002; Heidsieck et al., 2016; Finkelstein et al., 1990)	Trigeminal nerve (CN V3)	Tensor veli palatini (TVP)	Tensioning & tuning (modulating acoustic impedance)	Trigeminal nerve stimulation (TNS)
Visceral system (branchial arch–derived musculature) (Kuehn and Moon, 1998; Gyanwali et al., 2016; Perry, 2011)	Vagus Nerve (CN X) glossopharyngeal Nerve (CN IX)	Levator veli palatini (LVP) pharyngeal constrictors	Sealing & peristalsis (controlling aerodynamics)	Vagus nerve stimulation (VNS)

This table summarizes the principal cranial nerve innervation of key palatal muscles and their associated functional roles in oropharyngeal coordination. (TVP, tensor veli palatini; LVP, levator veli palatini; NTS, nucleus tractus solitarius; NA, nucleus ambiguus).

## Neuromotor regulation: brainstem CPGs and the reflex-volition coupling

**Figure 2:**
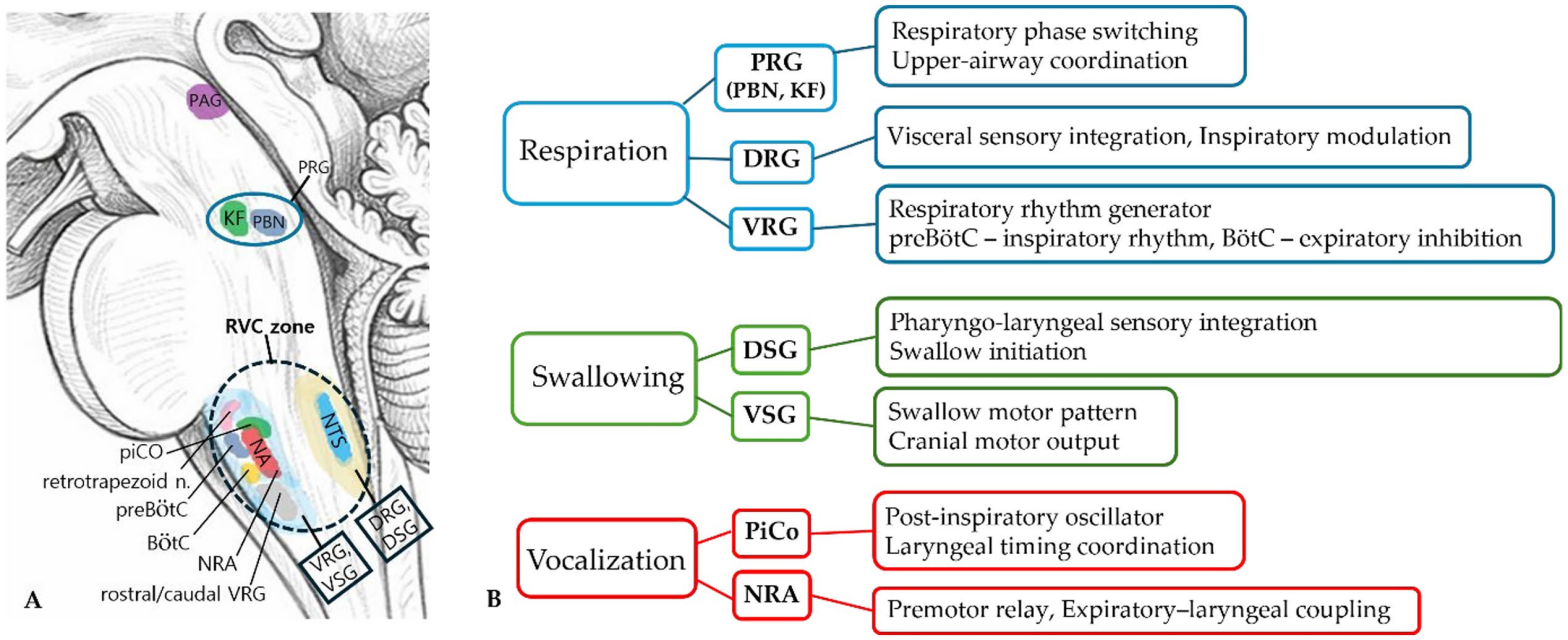
Brainstem circuits associated with the RVC zone. **(A)** Brainstem localization of the RVC zone: Sagittal schematic of the brainstem illustrating the approximate location of the RVC (reflex–volition coupling) zone within brainstem. The region lies between the nucleus tractus solitarius (NTS) and nucleus ambiguus (NA) and overlaps with respiratory and oropharyngeal premotor networks of the ventral respiratory column. **(B)** Major circuit components associated with the RVC zone: Summary of brainstem circuits involved in respiration, swallowing, and vocalization that functionally interact with the RVC region (Jean, 2001; Smith et al., 2007; Huff et al., 2023; Dutschmann and Dick, 2012; Ikeda et al., 2017; Pitts and Iceman, 2023). 1) Anatomical basis of swallowing: The swallowing network is functionally organized into a dorsal swallowing group (DSG) and a ventral swallowing group (VSG). The DSG corresponds largely to the central and interstitial subnuclei of the nucleus tractus solitarius (NTS) and integrates sensory inputs that initiate the swallowing sequence. The VSG is distributed within the nucleus ambiguus (NA) and the surrounding ventrolateral reticular formation, where it organizes and distributes patterned motor output to cranial motor nuclei controlling the pharyngeal musculature. 2) Brainstem control of vocalization: Unlike respiration or swallowing, vocalization does not rely on a single dedicated central pattern generator. Instead, brainstem premotor structures such as the post-inspiratory complex (PiCo) and the nucleus retroambiguus (NRA) interact with respiratory circuits to coordinate laryngeal and expiratory activity required for vocalization. Although the pontine respiratory group (PRG), including the parabrachial and Kölliker-Fuse nuclei, lies rostral to the RVC region, it is included here because of its well-established role in respiratory phase switching and upper-airway coordination, which indirectly influences laryngeal and pharyngeal motor control. (BötC, Bötzinger complex; DRG, dorsal respiratory group; DSG, dorsal swallowing group; KF, Kölliker-Fuse nucleus; NA, nucleus ambiguus; NRA, nucleus retroambiguus; NTS, nucleus tract solitarius; PAG, periaqueductal gray; PBN, parabrachial nucleus; PiCo, post-inspiratory complex; preBötC, pre-Bötzinger complex; PRG, pontine respiratory group; VRG, ventral respiratory group; VSG, ventral swallowing group).

The rhythmic automaticity of the PPC—manifesting as respiration, deglutition, and coughing—persist even under conditions of limited cortical involvement. This relative autonomy is generally attributed to brainstem CPG circuits within the medulla oblongata. Consistent with classic neurophysiological studies on swallowing control, the core of this circuitry relies on reciprocal connectivity between the NTS, which serves as the primary sensory processing station, and NA, which functions as a key motor output center (Jean, 2001; Jean, 1984). This NTS-NA loop contributes to rhythm generation and the coordination of sequential muscle activities essential for life support (Feldman and Del Negro, 2006; Smith et al., 2007). Given its phylogenetic lineage derived from the branchial arch apparatus, this intrinsic circuitry can be discussed in the context of a branchial-arch–derived CPG network, maintaining homeostasis with limited dependence on higher cortical control (Bass and Chagnaud, 2012; Bouchard et al., 2013). The principal brainstem circuits relevant to swallowing, respiration, and vocalization, together with their approximate relationship to the proposed RVC zone, are summarized in Figure 2 (Jean, 2001; Smith et al., 2007; Huff et al., 2023; Dutschmann and Dick, 2012; Ikeda et al., 2017; Pitts and Iceman, 2023).

**Figure 3:**
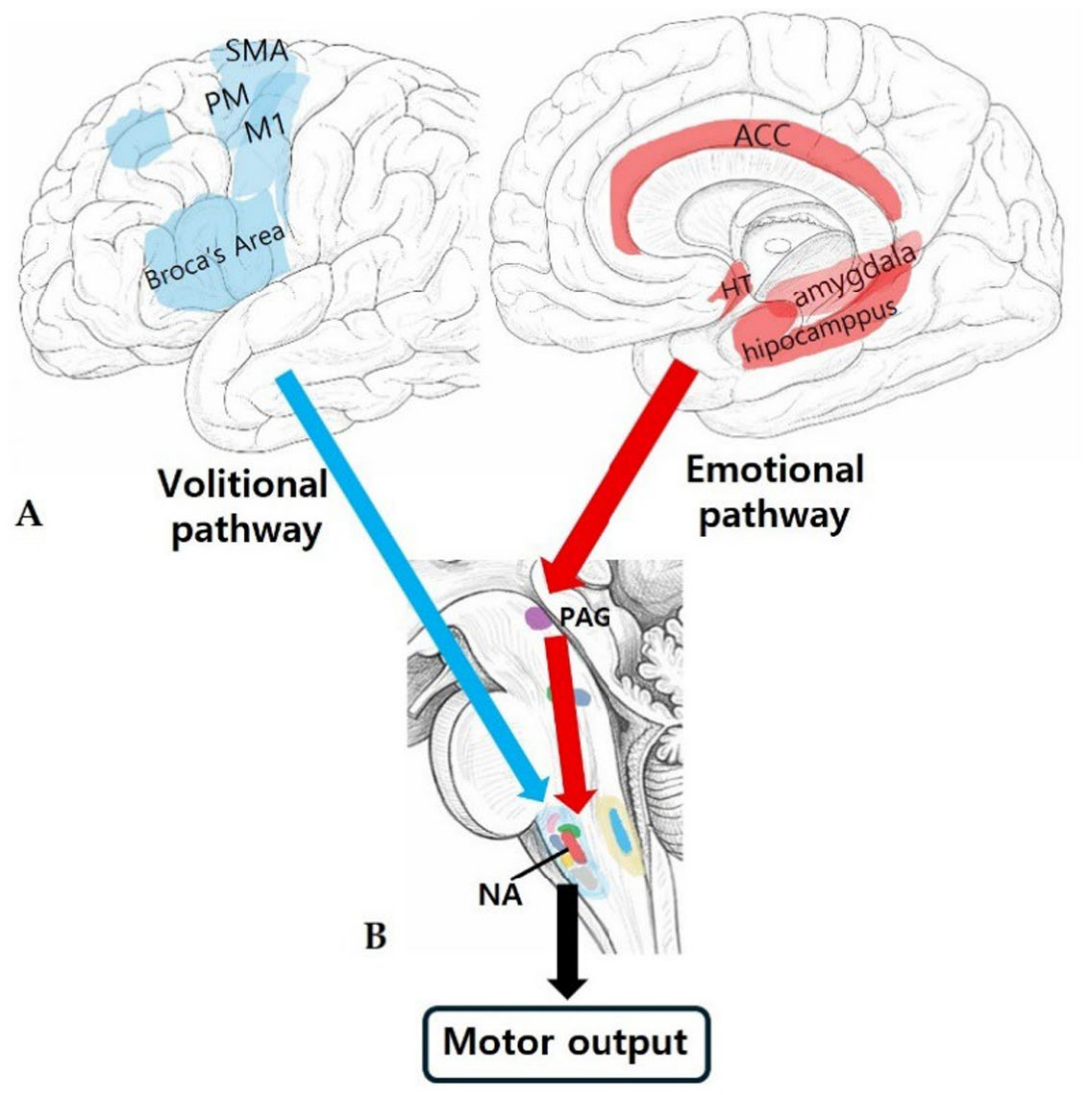
Simplified supranuclear pathways projecting to the RVC zone. **(A)** Cortical and limbic regions contributing to supranuclear vocal control: Schematic depiction of cortical motor areas involved in volitional vocalization (laryngeal motor cortex, premotor cortex, supplementary motor area) and limbic regions associated with emotional vocalization (anterior cingulate cortex, amygdala, hypothalamus). **(B)** Brainstem convergence toward the RVC zone: Sagittal brainstem schematic illustrating how descending inputs from these supranuclear systems converge toward the RVC zone, ultimately influencing premotor circuits and laryngeal motor output through the nucleus ambiguous (Simonyan, 2014; Jürgens, 2002; Hickok et al., 2023; Holstege et al., 1996; Schottelkotte and Crone, 2022). Vocalization is regulated by two major supranuclear control systems: volitional and emotional pathways. Volitional vocalization primarily originates from cortical motor areas, including the laryngeal motor cortex, premotor cortex, and supplementary motor area, whose descending corticobulbar projections influence brainstem premotor circuits. In contrast, emotional vocalization arises from limbic structures such as the anterior cingulate cortex, amygdala, and hypothalamus, and is relayed through the periaqueductal gray matter (PAG) before reaching the brainstem vocalization network. This figure schematically illustrates how these supranuclear systems converge toward the RVC zone, which ultimately drives laryngeal motor output. The detailed internal circuitry within the RVC zone is described in Figure 4. (ACC, anterior cingulate cortex; HT, hypothalamus; M1, primary motor cortex; NA, nucleus ambiguus; PAG, periaqueductal gray; PM, premotor cortex; SMA, supplementary motor area).

In humans, pharyngeal function often extends beyond purely reflexive control; it is dynamically modulated to accommodate higher-order behaviors. Building upon the “dual-pathway” models proposed by Jürgens (2002) and Holstege (1992), previous models suggest that the brainstem motor nuclei receive descending projections not only from the CPG but also from the limbic system (e.g., periaqueductal gray, anterior cingulate cortex) for emotional vocalization (Holstege, 1992; Jürgens, 2002), and from the neocortex (e.g., primary motor cortex, Broca’s area) for volitional speech and speech-related respiratory control (Simonyan and Horwitz, 2011; Moore et al., 2013). Recently, this conceptualization has been further discussed in evolutionary accounts, which suggest that human vocal communication relies on a dual motor coordination system that structurally segregates laryngeal emotional control from supralaryngeal articulatory control (Hickok et al., 2023). The simplified supranuclear pathways through which volitional and emotional influences may reach the brainstem vocalization network are illustrated in Figure 3 (Simonyan, 2014; Jürgens, 2002; Hickok et al., 2023; Holstege et al., 1996; Schottelkotte and Crone, 2022).

**Figure 4:**
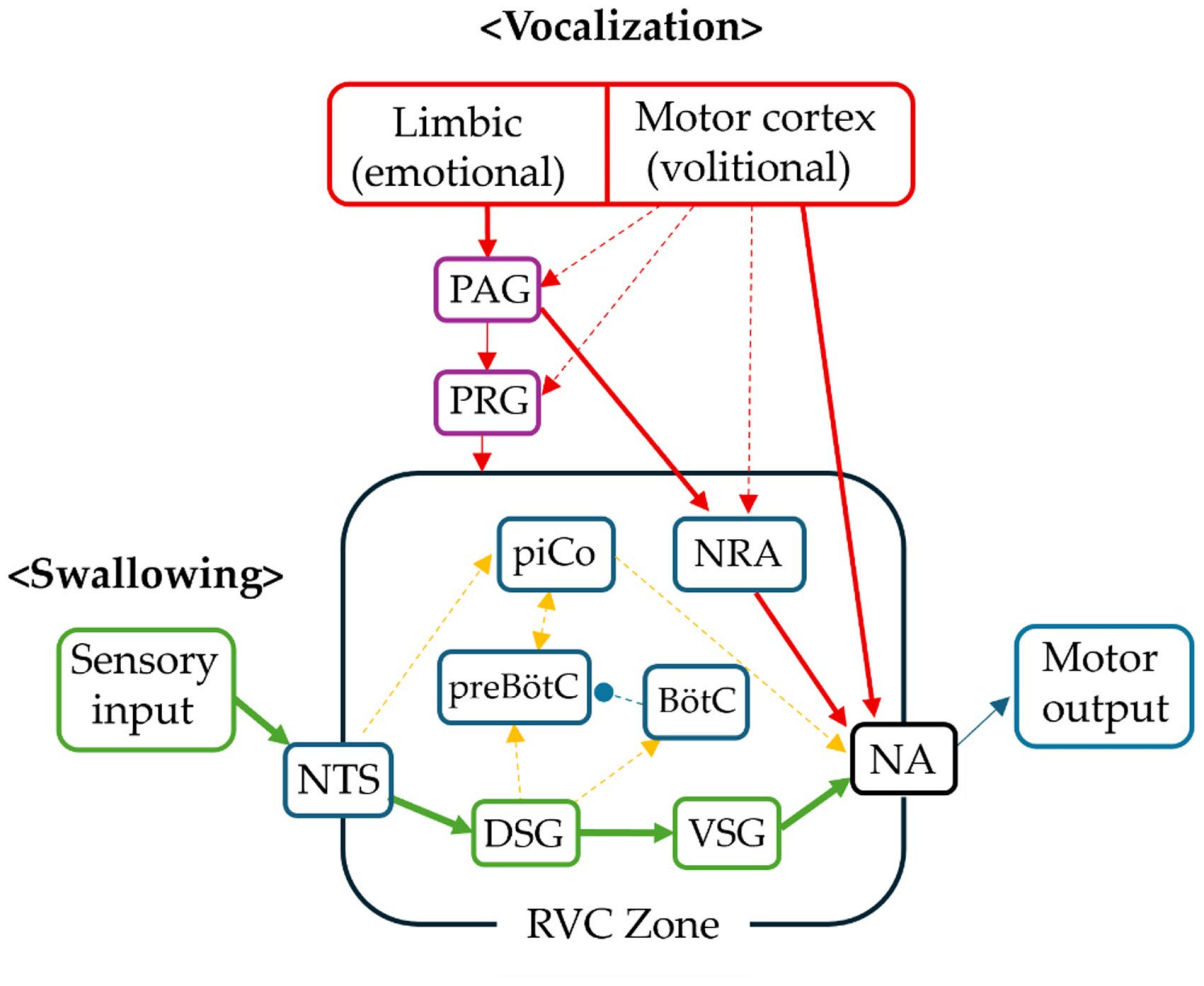
Conceptual integration of swallowing, respiration, and vocalization circuits within the RVC zone. This schematic illustrates the proposed interaction of swallowing, respiratory, and vocalization-related circuits within the RVC (reflex–volition coupling) zone, located in the brainstem. Swallowing reflexes are initiated when sensory inputs converge in the nucleus tractus solitarius (NTS) and activate the dorsal swallowing group (DSG). The patterned swallowing command is then distributed through the ventral swallowing group (VSG) to the nucleus ambiguus (NA) and other cranial motor nuclei, generating coordinated contractions of the pharyngeal and laryngeal musculature. Respiratory rhythm generation is centered in the ventral respiratory column, particularly the pre-Bötzinger complex (preBötC) and Bötzinger complex (BötC), which together shape inspiratory and expiratory respiratory patterns. Vocalization does not rely on a dedicated standalone central pattern generator. Instead, it recruits overlapping premotor circuits within the respiratory network. In particular, the nucleus retroambiguus (NRA) functions as a major premotor relay linking expiratory drive to laryngeal motor output through the nucleus ambiguus (NA). Supranuclear control reaches the RVC zone through two principal pathways. The emotional vocalization pathway originates in limbic structures and is relayed through the periaqueductal gray (PAG) and pontine respiratory group (PRG). In contrast, the volitional pathway arises from cortical motor areas and descends via corticobulbar projections to influence brainstem premotor circuits and NA motor neurons. In this schematic, solid arrows represent relatively well-established functional pathways, whereas dashed arrows indicate proposed or partially characterized interactions within the brainstem network. Within this framework, the RVC zone may act as an integrative field coordinating the timing and interaction of swallowing, respiration, and vocalization within the brainstem (Jean, 2001; Smith et al., 2007; Huff et al., 2022; Huff et al., 2023; Dutschmann and Dick, 2012; Paydarfar et al., 1995; Moreira et al., 2025). (BötC, Bötzinger complex; DSG, dorsal swallowing group; NA, nucleus ambiguus; NRA, nucleus retroambiguus; NTS, nucleus tract solitarius; PAG, periaqueductal gray; PiCo, post-inspiratory complex; preBötC, pre-Bötzinger complex; PRG, pontine respiratory group; VSG, ventral swallowing group).

This architecture suggests a hierarchical organization in which the nervous system may need to prioritize competing commands: immediate airway protection (brainstem level) may constrain emotional expression (limbic level), which in turn interacts with volitional control (cortical level) (Dutschmann and Dick, 2012; Paydarfar et al., 1995). For instance, while cortical commands can temporarily suppress respiration for speech, homeostatic drive may override such suppression under increasing homeostatic drive, demonstrating the survival-first hierarchy of this arbitration.

To conceptualize the functional interface where descending volitional and affective signals intersect with ascending reflexive rhythms, we use the term “RVC zone” to refer to a putative brainstem interface associated with the reticular formation between the NTS and NA. Within this framework, the RVC zone may be considered in terms of two functional roles: gating and timing coordination (Kleinfeld et al., 2014; Hickok et al., 2023).

First, inhibitory interactions are implicated during the “swallow apnea” phase, where the activation of the swallowing CPG transiently inhibits the respiratory and phonatory circuits to prevent aspiration (Paydarfar et al., 1995; Jean et al., 1975). Second, coordination is required during vocalization, where cortical volitional commands must precisely phase-lock with the expiratory phase of the respiratory CPG to produce sound (McFarland and Smith, 1992; McFarland, 2001). Thus, the proposed RVC zone is discussed as a functional interface that may influence the timing and gain of brainstem reflex activity in the context of voluntary and affective control. As a conceptual synthesis of these relationships, Figure 4 illustrates how swallowing, respiratory, and vocalization-related circuits may interact within the proposed RVC zone (Jean, 2001; Smith et al., 2007; Huff et al., 2022; Huff et al., 2023; Dutschmann and Dick, 2012; Paydarfar et al., 1995; Moreira et al., 2025).

Consequently, this perspective raises the possibility that clinical conditions such as dysphagia or functional dysphonia may reflect, at least in part, altered interactions between supranuclear inputs and brainstem patterning networks (Gracco and Lofqvist, 1994; Hamdy et al., 1996). This hierarchical arbitration of neural commands is summarized in Table 2 (Jean, 2001; Smith et al., 2007; Huff et al., 2022; Rygula et al., 2012; Holstege, 1992; Dutschmann and Dick, 2012; Ikeda et al., 2017; Jürgens, 2002; Holstege et al., 1996).

**Table 2:** Hierarchical levels of neural control in the pharyngeal axis.

Control level	Primary function	Neural substrate	Interaction at RVC zone
Level 1: reflexive (metabolic) (Jean, 2001; Smith et al., 2007; Huff et al., 2022)	Life support (swallowing, breathing)	Brainstem CPGs (NTS—nucleus ambiguus loop)	Reciprocal inhibition (gating) (e.g., swallowing suppresses respiration)
Level 2: Affective (Limbic) (Rygula et al., 2012; Holstege, 1992; Dutschmann and Dick, 2012)	Emotional expression (laughing, crying)	Limbic system (PAG, anterior cingulate cortex)	Emotional Modulation (Limbic drive transiently modulates respiratory rhythm)
Level 3: volitional (cognitive) (Ikeda et al., 2017; Jürgens, 2002; Holstege et al., 1996)	Communication (speech, breath-holding)	Neocortex (M1, SMA, Broca’s area)	Cooperative synchronization (solitional onset phase-locks with CPG)

The table outlines brainstem, limbic, and cortical contributions to pharyngeal motor regulation and illustrates how these levels may interact within the proposed Reflex-Volition Coupling framework. CPG, central pattern generator; NTS, nucleus tractus solitarius; NA, nucleus ambiguus; PAG, periaqueductal gray; ACC, anterior cingulate cortex; M1, primary motor cortex; RVC, Reflex-Volition Coupling).

## Functional implications of the palato-pharyngeal complex within brainstem integrative networks

In addition to its role as a peripheral structure involved in mechanical rehabilitation, the PPC may also be considered a dense sensory interface whose afferent projections engage brainstem networks involved in adaptive coordination and plasticity. However, emerging evidence from non-invasive brain stimulation studies, such as rTMS (repetitive transcranial magnetic stimulation) and tDCS (transcranial direct current stimulation), suggests that functional recovery may involve neuroplastic reorganization of cortico-bulbar connectivity in addition to peripheral muscular adaptation (Hamdy et al., 1998; Pisegna et al., 2016).

The pharyngeal mucosa is densely innervated by visceral afferents of the glossopharyngeal and vagus nerves, which project directly to the NTS. Sensory stimulation of the pharyngo-laryngeal mucosa is known to trigger airway protective reflexes such as swallowing, coughing, and transient apnea (Yoshida et al., 2000). Mechanical and chemical stimulation of these regions activates glossopharyngeal and vagal afferents that converge in the NTS and interact with brainstem swallowing and respiratory networks (Lang, 2009). These reflex circuits play a key role in coordinating swallowing with respiration and maintaining airway protection. Recent studies further indicate that distinct vagal sensory neuron populations mediate chemosensory detection within the laryngo-pharyngeal epithelium, suggesting potential mechanisms through which peripheral sensory input may influence brainstem integrative circuits (Prescott et al., 2020; Soma et al., 2025).

Within this framework, patterned sensory afferents arising from the PPC may influence activity in NTS-centered brainstem networks, as proposed in the RVC model (Fraser et al., 2002). The NTS maintains extensive reciprocal connections with the locus coeruleus and reticular formation, regions involved in the regulation of arousal, attention, and autonomic balance (Aston-Jones and Cohen, 2005; Berntson and Khalsa, 2021). This widespread integrative role is strongly supported by recent human connectome data detailing the extensive structural and functional connectivity of the NTS to key cortical and autonomic regions (Forstenpointner et al., 2022). Moreover, state-of-the-art neuroimaging demonstrates that brainstem-driven rhythmic activities, such as respiration, selectively modulate brain-wide functional connectivity, linking physiological CPGs directly to large-scale cognitive and emotional networks (Mohammadi et al., 2025). While current transcutaneous vagus nerve stimulation (tVNS) approaches mainly target the auricular branch of the vagus nerve (Butt et al., 2020), the PPC represents an anatomically distinct site where somatic and visceral afferents converge. This convergence raises the possibility that intra-oral sensory pathways may engage multiple brainstem integrative circuits (Yates and Stocker, 1998; Steele and Miller, 2010). Whether stimulation of this region produces functional effects distinct from other neuromodulation routes remains to be determined. Nonetheless, early clinical studies using pharyngeal electrical stimulation indicate that targeting pharyngeal afferents can influence swallowing-related neural networks (Jayasekeran et al., 2010; Bath et al., 2016). A comparative overview of these neuromodulation approaches is summarized in Table 3 (Fraser et al., 2002; Forstenpointner et al., 2022; Mohammadi et al., 2025; Jayasekeran et al., 2010; Bath et al., 2016; Johnson and Wilson, 2018).

**Table 3:** Neuromodulation approaches engaging brainstem afferent pathways.

Modulatory approach	Primary target	Mechanism	Potential application
Cervical VNS (invasive) (Johnson and Wilson, 2018)	Cervical vagus nerve trunk	Direct depolarization of parasympathetic fibers	Epilepsy, Depression (FDA approved)
Auricular tVNS (non-invasive) (Soma et al., 2025; Steele and Miller, 2010)	Auricular branch (Ear)	Indirect activation of NTS via cutaneous afferents	Migraine, pain
Intra-oral PPC stimulation (proposed) (Berntson and Khalsa, 2021; Forstenpointner et al., 2022; Jayasekeran et al., 2010)	Palato-pharyngeal plexus (V, IX, X convergence)	Multi-modal engagement (simultaneous somatic & visceral activation)	Dysphagia rehabilitation, arousal regulation, cortical plasticity

This table compares different stimulation strategies that engage cranial nerve afferents and their potential influence on brainstem integrative circuits. (VNS, vagus nerve stimulation; tVNS, transcutaneous vagus nerve stimulation; rTMS, repetitive transcranial magnetic stimulation; tDCS, transcranial direct current stimulation; PPC, Palato-Pharyngeal Complex).

## Conclusion

This review discusses the PPC as an anatomically integrated region where cranial nerve afferents converge and interact with brainstem circuits involved in oropharyngeal coordination. By outlining a conceptual framework that links reflexive brainstem patterning with supranuclear influences, we aim to provide a coherent perspective for interpreting the organization and regulation of palato-pharyngeal behaviors. Further work combining detailed neuroanatomical mapping with physiological and behavioral approaches will be important to clarify how oropharyngeal sensory pathways engage brainstem and cortical networks and how these interactions may be leveraged in rehabilitation and neuromodulation contexts (Simons and Hamdy, 2017; Teismann et al., 2009; Suntrup et al., 2015; Sclocco et al., 2019; Dziewas et al., 2018).
